# EphB2 activation in neural stem cells in the basolateral amygdala facilitates neurogenesis and enhances long-term memory

**DOI:** 10.1007/s00018-024-05317-w

**Published:** 2024-06-24

**Authors:** Karishma Agarwal, Raphael Lamprecht

**Affiliations:** Sagol Department of Neurobiology, Faculty of Natural Sciences, University of Haifa, Haifa, Israel

**Keywords:** Long-term memory, Amygdala, Neural stem cells, EphB2

## Abstract

Many brain diseases lead to a reduction in the number of functional neurons and it would be of value to be able to increase the number of neurons in the affected brain areas. In this study, we examined whether we can promote neural stem cells to produce mature neurons and whether an increase in the mature neurons can affect cognitive performance. We detected that the EphB2 receptor is localized in immature basolateral amygdala (BLA) neurons. We therefore aimed to increase the level of EphB2 activity in neural stem cells (NSCs) in the BLA and examine the effects on the production of mature neurons and cognition. Toward that end, we utilized a photoactivatable EphB2 construct (optoEphB2) to increase EphB2 forward signaling in NSCs in the BLA. We revealed that the activation of optoEphB2 in NSCs in the BLA increased the level of immature and mature neurons in the BLA. We further found that activation of optoEphB2 in BLA NSCs enhanced auditory, but not contextual, long-term fear memory formation. Impairing EphB2 forward signaling did not affect the level of immature and mature neurons in the BLA. This study provides evidence that NSCs can be promoted to produce mature neurons by activating EphB2 to enhance specific brain functions.

## Introduction

Neurogenesis is involved in cognitive processes and many neurological and psychiatric disorders involve a reduction in neurogenesis in the affected brain areas [1, 2]. The majority of studies that examined the relationship between neurogenesis and cognition were performed in the hippocampus. For example, neurogenesis is correlated with behavioral performance where increased neurogenesis in the hippocampus (e.g. by enriched environment) is associated with improved hippocampus-dependent behavior. In contrast, reduced neurogenesis (in aged mice) is associated with impairments in hippocampus-dependent learning tasks [1]. In addition, studies have shown a causal role of adult neurogenesis in the generation or modification of specific behaviors. For example, the blockade of neurogenesis in the adult mouse by an antimitotic agent disrupts trace eye-blink conditioning and fear conditioning [3, 4]. Neurogenesis can also be found in the adult amygdala [4–8].

The above studies suggest that the formation of new functional neurons from NSCs can be beneficial for the treatment of brain diseases and improve cognitive performance. In this study, we examined whether activation of EphB2 can serve this purpose. EphB2 is a tyrosine kinase receptor that is activated by its ephrin cognate ligands [9]. EphB2 dysfunction impairs neurogenesis in the hippocampus of adults. For example, mice lacking both EphB1 and EphB2, have significantly fewer neural progenitors in the hippocampus [10] and EphB2 impaired in forward signaling reduces DCX-positive late-stage progenitors in the hippocampus in mice [11]. Another study has shown that EphB2 is essential to sustain NSC quiescence [12]. Thus, EphB2 function in NSCs is involved in regulating neurogenesis in the adult hippocampus. In this study, we aim to test whether activation of EphB2 in NSCs leads to the formation of new mature neurons and whether it affects cognition.

To investigate whether the activation of EphB2 in NSCs leads to the facilitation of the production of mature neurons and whether it can affect memory formation we use in the current study an optogenetic approach to activate EphB2 forward signaling by light (optoEphB2) [13, 14]. This technique takes advantage of the fact that EphB2 activation is induced by receptor clustering [15]. In optoEphB2, the EphB2 cytoplasmic domain is fused to an optimized mutant of the plant protein cryptochrome 2 (Cry2olig, residues 1–498 with E490G mutation) which is clustered by blue light [16]. OptoEphB2 includes also mCherry for in vivo detection and is myristoylated at the N-terminus to be directed to the membrane. We have shown that stimulating of optoEphB2 by light in the brain leads to its activation (formation of receptor clusters and phosphorylation) and the activation of downstream molecular signaling (phosphorylation of proteins including Src) [14]. Thus, the application of blue light leads to EphB2 clustering and activation. This technique allows us to activate EphB2 at the highest required spatiotemporal resolution in vivo.

To express optoEphB2 in NSCs, we used a floxed optoEphB2 in adeno-associated virus (AAV). We microinjected the AAV into Nestin-Cre^ERT2^ mice and tested whether its activation by light can enhance the ability of Nestin^+^ NSCs to produce mature neurons. To examine whether EphB2 activation in NSCs can facilitate memory formation we used auditory fear conditioning, a well-established behavioral paradigm. In this paradigm, an association is formed between the auditory stimulation (conditioned stimulus (CS)) and an aversive mild footshock (unconditioned stimulus (US)) [17–21]. The putative site of fear conditioning memory, the basolateral amygdala (BLA), has been identified [17–20, 22–24]. We detected the localization of EphB2 in DCX immature neurons in the BLA. We, therefore, aimed to increase the level of EphB2 activity in NSCs in the BLA by optoEphB2 and examine the effects on mature neuronal production and the formation of fear conditioning long-term memory.

## Materials and methods

### Animals

Adult males 8 weeks of age were used. C57BL/6-Tg(Nes-cre/ERT2)KEisc/J (The Jackson Laboratory) were injected with 4-hydroxytamoxifen (4-OHT; Sigma-Aldrich, Cat#H6278) (i.p. 50mg/kg of 5mg/ml) once a week before the experiment. The generation of EphB2^lacZ/lacZ^ has been described previously (Henkemeyer et al., 1996). Colonies founder mice were obtained from Prof. Ruediger Klein and were bred at the University of Haifa. Mice were housed at 22°C in a 12h light/dark cycle, with free access to food and water. The experiments were approved by the University of Haifa Institutional Committee for animal experiments in accordance with the National Institutes of Health guidelines.

### AAV production

OptoEphB2 was obtained from Ji Yu (University of Connecticut Health Center, USA). Cloning and AAV production were performed at the ELSC Vector Core Facility (Hebrew University of Jerusalem, Israel). Double-floxed inverse open reading frame (DIO) optoEphB2 under the control of CBh promoter was created (AAV9-CBh-DIO-optoEphB2-mCherry) at a titer of 5.0 E + 13.

### AAV microinjection

Animals were anesthetized with Medetomidine (Domitor) 1mg/ml and Ketamine 100mg/ml cocktail, diluted in sterile isotonic saline (administered doses: Ketamine 50mg/kg; Domitor 0.5mg/kg; 100µl/10gm of animal body weight). Dipyrone (50%) was injected for analgesia before surgery and consecutive 3 days after surgery. AAV particles were injected (0.5µl/hemisphere, 0.1µl/min) aimed at the BLA. The coordinates for performing stereotactic surgery were measured according to the mouse bregma location in the Mouse brain atlas (coordinates are in reference to bregma: anteroposterior (AP), − 1.4; lateral (L) ± 3.3; and dorsoventral (DV), − 4.65). Animals were allowed to recuperate for 4 weeks before behavioral experiments. After behavioral or histological procedures, the animals were perfused and the localization of AAVs was examined. Only mice with expression of mCherry within the borders of the BLA were included in the data analysis.

### Optic fiber implantation and laser stimulation

For optogenetic experiments, intracranial optic fibers (Thorlabs, Fiber Optic Cannula, Ø1.25 mm Stainless Ferrule, Ø200 µm Core, 0.39 NA) were implanted at the following coordinates: AP, – 1.4, L, ± 3.3, DV, 4.15 (relative to bregma). Optic fibers were connected to a 473-nm blue laser diode (Shanghai Dreamlasers) via FC/PC adaptors. The light intensity measured at the tip of the fiber was approximately 15mW/mm^2^. After 4-OHT administration, the mice were allowed to recover for a week. Following recovery, the laser stimulation was applied for three consecutive days and followed a 9 min protocol. Animals were placed in the conditioning chamber and two minutes afterward subjected to laser stimulation thrice for 20 s each with an inter-trial interval of 120 s.

### Fear conditioning

On the day of training, mice were placed in a training chamber (Coulbourn Instruments). Mice were allowed to acclimate in the chamber for 2 min and then subjected to 3 pairs of tone (Conditioned stimulus (CS)—20 s, 2.8 kHz, 85 dB) that co-terminated with a foot shock (Unconditioned stimulus (US)—2 secs, 0.8 mA). The inter-trial interval was 120 s. Mice were tested for contextual fear conditioning long-term memory in the same context (for 9 min) 24 h after training. Mice were tested for auditory fear conditioning long-term memory in a different context 48 h after training. Behavior was recorded and the video images were transferred to a computer equipped with an analysis program. The percentage of changed pixels between two adjacent 0.25 s images was used as a measure of activity.

### Immunohistochemistry

Animals were anesthetized by inhalation of isoflurane and transcardially perfused with 50 ml of cold 0.01 M PBS solution, followed by 50 ml of 4% paraformaldehyde in 0.01 M PBS. Brains were excised and postfixed in a fixative solution containing 30% sucrose and 1% paraformaldehyde in 0.01 M PBS for 48 h at 4°C. After postfixation, brains were frozen at – 80°C until sectioning. Forty-micrometer brain sections were sliced with a cooled cryostat (Leica, CM1900). Slices were washed with 0.01 M PBS and then blocked for 1 h at room temperature with 0.01 M PBS containing 3% BSA. Sections were then incubated overnight at 4°C with anti-DCX (Cell Signaling Technology, #4604; 1:800), anti-Nestin (Sigma-Aldrich #MAB353; 1:100) or anti-NeuN (Sigma-Aldrich #ABN78; 1:500). Next, after three washes in 0.01 M PBS, the slices were subjected to Alexa-488 anti-rabbit or Alexa-488 anti-mouse secondary antibody (1:500 Molecular Probes) for 1.5 h at room temperature. The slices were then washed twice with PBS 0.01 M and mounted on Super Frost-coated slides with Slow Fade antifade medium (Invitrogen). The level of labeling was calculated using the Imaris software.

For GFAP staining brain sections were permeabilized and blocked in 0.01 M PBS solution containing 0.5% Triton X-100 and 10% normal donkey serum (NDS) for 45 min at room temperature on a shaker. Subsequently, brain sections were incubated with Anti-GFAP (1:200, CST-3670S, Cell Signaling Technologies) in 0.01 M PBS solution containing 2% NDS overnight at 4°C on a shaker. The following day, the sections were washed thrice for 5 min each with the 0.01 M PBS solution and incubated with fluorescently labeled secondary anti-Mouse Alexa Fluor 647 (1:200, A-21235, Invitrogen) in 0.01 M PBS solution containing 2% NDS overnight at 4°C on a shaker in a dark place. The next day, the sections were washed thrice for 5 min each with 0.01 M PBS solution. The sections were then mounted onto the glass slides with mounting medium and stored in a dark place until imaging.

### Statistics

Data were analyzed with repeated measures ANOVA for multiple tone presentation behavioral analysis and with non-parametric Mann–Whitney U test for contextual fear conditioning and IHC experiments with an α level of 0.05. To correlate between individual mouse percentage freezing and the number of DCX or NeuN stained cells we performed Pearson correlation. These analyzes were performed using the PASW statistics 25.

## Results

### Activation of EphB2 in neural stem cells in the BLA facilitates neurogenesis and the formation of mature neurons

**Fig. 1:**
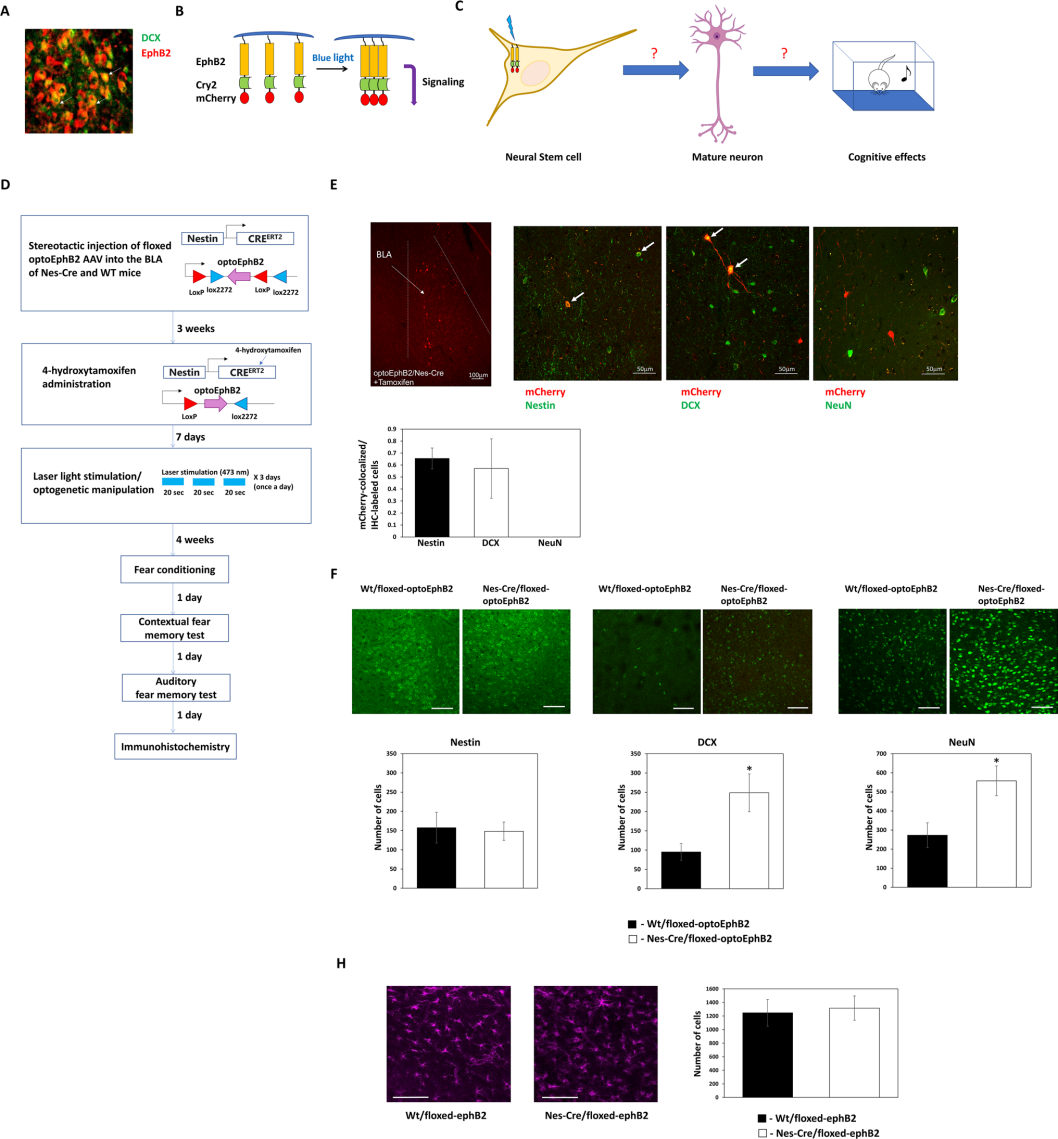
EphB2 activation in neural stem cells in BLA facilitates neurogenesis and the formation of mature neurons. **A** EphB2 receptor is localized in immature neurons expressing DCX. Arrows show examples of EphB2 and DCX co-labeled cells. **B** To activate EphB2 forward signaling in cells we have used the photoactivatable EphB2 (optoEphB2) that forms clusters upon light stimulation through Cry2olig. The formation of such clusters leads to optoEphB2 forward signaling activation. **C** In the study, we asked whether activation of EphB2 forward signaling in neural stem cells (NSCs) in BLA will lead to the formation of mature neurons in BLA and affect long-term fear memory formation. **D** AAV containing floxed-optoEphB2 construct were injected into C57BL/6-Tg(Nes-cre/ERT2)KEisc/J mice BLA. After 3 weeks the mice were administered with 4-hydroxy tamoxifen once at 5 mg/ml to express the optoEphB2. Control animals were wt littermate animals injected with the floxed-optoEphB2 and subjected to 4-OHT and light protocol but did not express optoEphB2. Seven days later, optoEphB2 expressed in Nestin-expressing stem cells was activated by light (3 times 20 s with ITI of 120 s) once a day for 3 days. Four weeks later, the mice were trained for fear conditioning and tested for long-term fear memory. The next day the brains were subjected to immunohistochemistry. **E** OptoEphB2 is expressed, after administration of 4-OHT and immediately after the 3 days of light activation, in the BLA in Nestin and DCX-labeled cells but not NeuN-labeled cells. The portion of the colocalized cells expressing optoEphB2 (mCherry) from the total labeled cells examined is shown. **F** Representative figure of Nestin, DCX and NeuN immunohistochemical staining in BLA is shown. Activation of optoEphB2 (n = 8) did not affect the number of Nestin-expressing cells when compared to animals where optoEphB2 was not expressed (n = 5) (t_(11)_ = 0.217; p = 0.833). Activation of optoEphB2 (n = 6) increased the number of DCX-labeled cells in BLA when compared to animals that did not express optoEphB2 (n = 5) (t_(9)_ = 2.671, p = 0.026). Activation of optoEphB2 (n = 10) increased the number of NeuN-expressing neurons compared to animals that did not express optoEphB2 (n = 9) (t_(17)_ = 2.759, p = 0.013). **H** There is no change in the number of GFAP-labeled astrocytes between the groups (t_(6)_ = 0.261, p = 0.803)

We observed that EphB2 is localized in immature neurons in the BLA as seen by its co-localization with cells expressing DCX (Fig. 1A). We were therefore interested in exploring whether activation of EphB2 forward signaling in neural stem cells (NSCs) in the BLA will affect the formation of mature neurons and memory formation. Toward that end, we used an optogenetic approach to activate EphB2 forward signaling by light (optoEphB2) [13–15] (Fig. 1B). We asked whether the activation of optoEphB2 in NSCs will facilitate the formation of mature neurons in the BLA (Fig. 1C). Toward that end, we injected AAV containing a floxed-optoEphB2 construct (photoactivatable EphB2) into C57BL/6-Tg(Nes-cre/ERT2)KEisc/J mice BLA. After 3 weeks, the mice were administered with 4-OHT to express optoEphB2 in NSCs (Fig. 1D). Control animals were littermate wild-type (wt) animals injected with the floxed-optoEphB2 AAV and subjected to 4-OHT and the light protocol. As can be seen after the administration of 4-OHT, a period of 7 days and subsequent 3 days of light activation, optoEphB2 is expressed in the BLA in Nestin and DCX cells, but not NeuN cells, indicating that the light application affects optoEphB2 only in NSCs and immature neurons (Fig. 1E). In another set of experiments, optoEphB2 expressed in Nestin-expressing NSCs was activated with light (3 times for 20 s each with ITI of 120 s) once a day for 3 days. Four weeks later, the mice were trained for fear conditioning and tested for long-term fear memory. We then examined the effects of activating optoEphB2 in NSCs on the amounts of NSCs, immature neurons and mature neurons by monitoring Nestin, DCX and NeuN labeled cells in the BLA, respectively. We found that activation of optoEphB2 (n = 8) did not affect the number Nestin expressing cells when compared to animals where optoEphB2 was not expressed (n = 5) (t_(11)_ = 0.217; p = 0.833) (Fig. 1F). Activation of optoEphB2 (n = 6) increased the number of DCX-labeled cells in BLA when compared to animals that did not express optoEphB2 (n = 5) (t_(9)_ = 2.671, p = 0.026) (Fig. 1F). Activation of optoEphB2 (n = 10) increased the number of NeuN-expressing neurons compared to animals that did not express optoEphB2 (n = 9) (t_(17)_ = 2.759, p = 0.013) (Fig. 1F). There is no change in the number of GFAP-labeled astrocytes between the groups (t_(6)_ = 0.261, p = 0.803) (Fig. 1H). Thus, activation of EphB2 forward signaling in NSCs leads to the increased production of immature and mature neurons in the BLA.

### Increasing EphB2 forward signaling activity in neural stem cells in the BLA enhances long-term fear memory

**Fig. 2:**
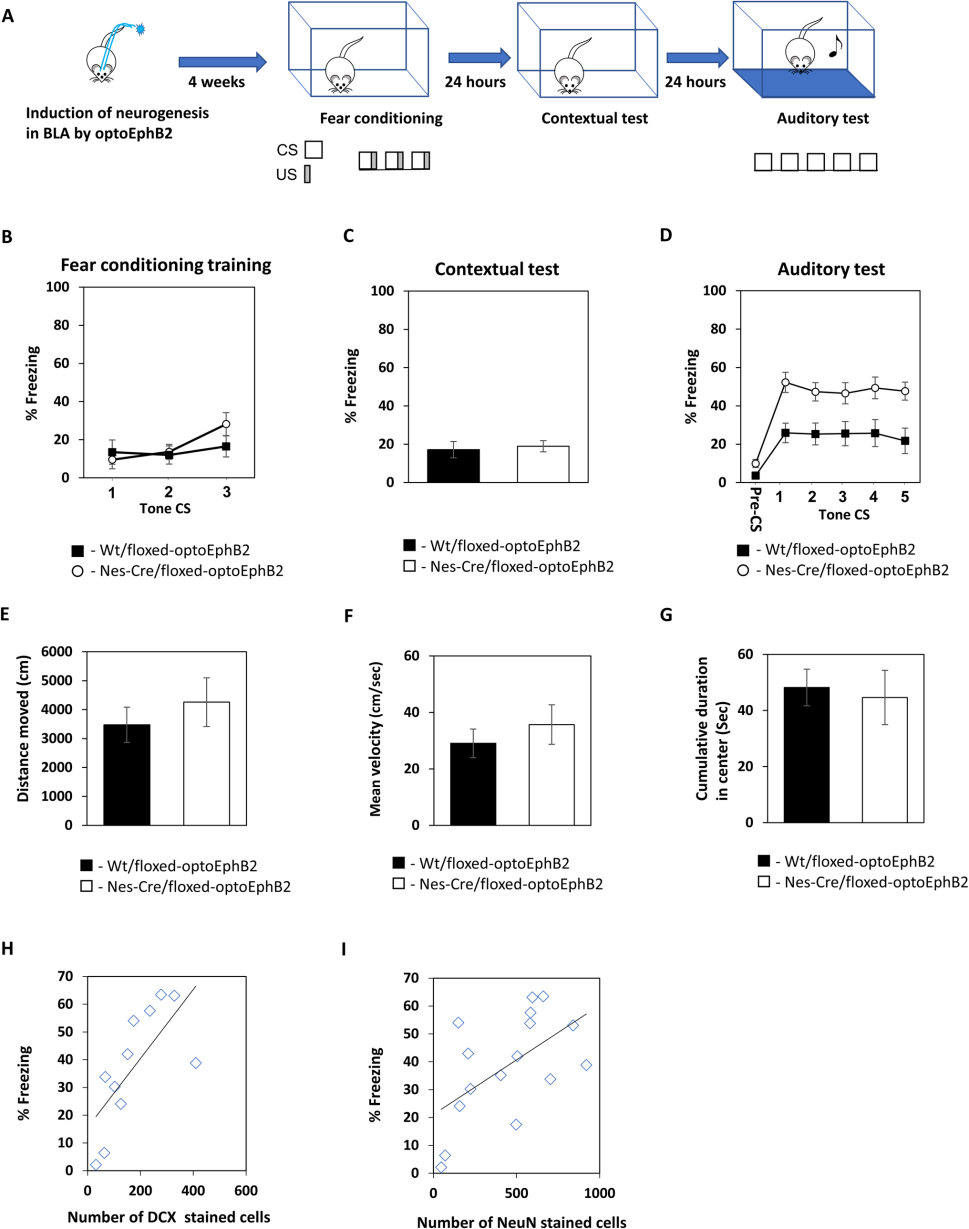
Increasing EphB2 forward signaling activity in neural stem cells in BLA enhances long-term fear memory. **A** Mice expressing the optoEphB2 in NSCs in BLA were stimulated by light. One month later the mice were fear conditioned. Twenty-four hours after training the animals were tested for contextual fear conditioning memory and 48 h after training for auditory fear conditioning memory. **B** There is no difference in freezing in training during the tones (F_(1,19)_ = 0.282, p = 0.601) and no tone X treatment interaction (F_(1.493,28.373)_ = 2.015, p = 0.161) **C** The optoEphB2-activated mice (n = 11) and control mice (n = 10) were not different when tested for contextual fear conditioning memory (p = 0.481). **D** Auditory fear conditioning memory in the animals where optoEphB2 is activated in NSCs (n = 11) was significantly enhanced when compared to control mice (n = 10) (F_(1,19)_ = 10.755; p = 0.004). **E** We see no difference between the groups in total distance movement (t_(19)_ = 0.743, p = 0.466) in the 2 min period before the fear conditioning training session. **F** We see no difference between the groups in mean velocity (t_(19)_ = 0.756, p = 0.459) in the 2 min period before the fear conditioning training session. **G** We see no difference between the groups in cumulative center chamber duration (t_(19)_ = 0.302, p = 0.766) in the 2 min period before the fear conditioning training session. **H** Long-term fear memory is positively correlated with the number of DCX-stained cells (n = 11; Pearson r = 0.708, p = 0.015). **I** Long-term fear memory is positively correlated with the NeuN-stained cells (n = 16; Pearson r = 0.568, p = 0.022)

The results above show that activation of optoEphB2 forward signaling by light in NSCs leads to an increase in mature neurons in the BLA. The BLA is essential for the formation of fear conditioning [17–23]. We were interested in exploring whether such an increase in the number of mature neurons after EphB2 activation affects the ability of the animals to form long-term fear memory formation. Toward that end, we injected AAV containing a floxed-optoEphB2 construct into C57BL/6-Tg(Nes-cre/ERT2)KEisc/J mice BLA. After 3 weeks, the mice were administered with 4-OHT to express optoEphB2 in NSCs. Control animals were littermate wt animals injected with the floxed-optoEphB2 AAV and subjected to 4-OHT and the light protocol. We activated optoEphB2 in NSCs 4 weeks before fear conditioning to allow the increase of mature neurons in BLA. We then trained the animals for fear conditioning and tested them for contextual fear conditioning memory 24 h after training and for auditory fear conditioning memory 48 h after training (Fig. 2A). There is no difference in freezing in training during the tones (F_(1,19)_ = 0.282, p = 0.601) and no tone X treatment interaction (F_(1.493,28.373)_ = 2.015, p = 0.161) (Fig. 2B). The optoEphB2-activated mice (n = 11) and control mice (n = 10) were not different when tested for contextual fear conditioning memory (p = 0.481) (Fig. 2C). However, auditory fear conditioning memory in the animals where optoEphB2 is activated in NSCs (n = 11) was significantly enhanced when compared to control mice (n = 10) (F_(1,19)_ = 10.755; p = 0.004) (Fig. 2D). There is no tone X treatment interaction (F_(2.671, 50.751)_ = 0.366; p = 0.755). To further characterize possible enduring effects of optoEphB2 on movement and anxiety we analyzed the movement of the animals, using Ethovision, in the fear conditioning chambers during the first 2 min before fear conditioning training. No difference between the groups was detected in total distance movement (t_(19)_ = 0.743, p = 0.466) (Fig. 2E), mean velocity (t_(19)_ = 0.756, p = 0.459) (Fig. 2F), and cumulative center chamber duration (t_(19)_ = 0.302, p = 0.766) (Fig. 2G). These results show that activation of optoEphB2 in the Nes-cre mice does not affect animal motor movement and anxiety. Further analysis shows that long-term fear memory is positively correlated with the number of DCX-stained (immature neurons) (n = 11; Pearson r = 0.708, p = 0.015) (Fig. 2H) and NeuN-stained cells (mature neurons) (n = 16; Pearson r = 0.568, p = 0.022) (Fig. 2I). These results show that activation of EphB2 forward signaling in NSCs increases the formation of neurons in the BLA and enhances the ability to form long-term fear memory in the BLA. Moreover, since optoEphB2 is active in stem cells and immature neurons but not mature cells it affects fear memory in these experiments through controlling immature cells and not mature neurons.

### EphB2 forward signaling dysfunction impairs the formation of fear conditioning long-term memory but not of immature or mature neurons in BLA

**Fig. 3:**
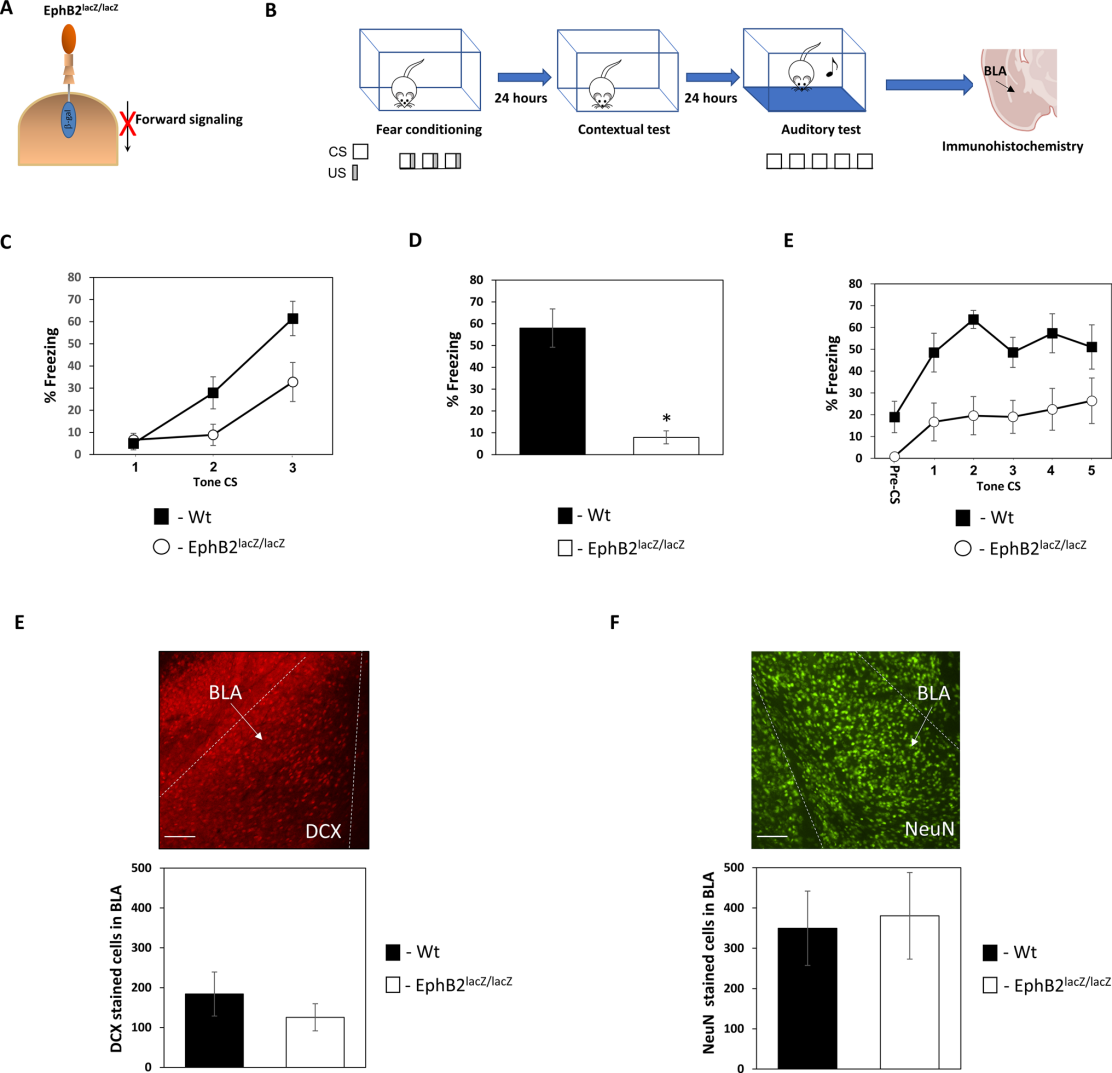
Impaired EphB2 forward signaling does not affect the formation of mature neurons in BLA. **A** To examine whether impairing EphB2 forward signaling affects the formation of immature and mature neurons in BLA we examined DCX and NeuN labeling in knockin mice that express mutant EphB2 (EphB2^lacZ/lacZ^) instead of the endogenous EphB2. In this mutant, the intracellular c-terminus of EphB2 contains β-gal instead of the normal c-terminus. The modified EphB2 is therefore impaired in forward signaling. **B** Animals were trained for fear conditioning and tested for long-term memory followed by immunohistochemistry to monitor the number of DCX-expressing immature neurons and NeuN-expressing mature neurons in the BLA. **C** The EphB2^lacZ/lacZ^ had different freezing responses from wt mice during training (F_(1,14)_ = 6.056, p = 0.027) with interaction (F_(2,28)_ = 4.057, p = 0.028). **D** EphB2^lacZ/lacZ^ (n = 9) mice were impaired in long-term contextual fear conditioning when compared to wild-type mice (n = 7) (p < 0.001). **E** EphB2^lacZ/lacZ^ (n = 9) mice were impaired in long-term auditory fear conditioning when compared to wild-type mice (n = 7) (F_(1,14)_ = 8.799, p = 0.01). **F** The BLA of these animals was stained with antibodies against DCX. There is no difference in the number of cells expressing DCX (EphB2^lacZ/lacZ^ (n = 9) and wildtype mice (n = 7)); p = 0.681). **G** The BLA of these animals was stained with antibodies against NeuN (EphB2^lacZ/lacZ^ (n = 9) and wildtype mice (n = 7)). There is no difference in the number of cells expressing NeuN (p = 0.791) in the BLA between wild-type and EphB2^lacZ/lacZ^ mice

The aforementioned results show that activation of EphB2 forward signaling in NSCs in BLA facilitates the formation of mature neurons and leads to their increase in the BLA. We were therefore interested in exploring whether impairing EphB2 forward signaling in the BLA will affect the formation of mature neurons. Toward that end, we studied neurogenesis in BLA of knockin mice (EphB2^lacZ/lacZ^) where an EphB2-lacZ (Nuk-LacZ) mutant allele, designed to encode a fusion protein, comprised of the extracellular, transmembrane and juxtamembrane domains but lacks the entire tyrosine kinase catalytic and C-terminal domains of EphB2, was introduced into mice by homologous recombination[25] (Fig. 3A). Animals were trained for fear conditioning and tested for long-term memory and the number of DCX and mature neurons in the BLA was monitored (Fig. 3B). The EphB2^lacZ/lacZ^ had different freezing responses from wt mice during training (F_(1,14)_ = 6.056, p = 0.027) with interaction (F_(2,28)_ = 4.057, p = 0.028) (Fig. 3C). EphB2^lacZ/lacZ^ (n = 9) mice were impaired in long-term contextual fear conditioning memory when compared to wild-type mice (n = 7) 24 h after training (p < 0.001) (Fig. 3D). EphB2^lacZ/lacZ^ (n = 9) mice were impaired in long-term auditory fear conditioning memory when compared to wild-type mice (n = 7) 48 h after training (F_(1,14)_ = 8.799, p = 0.01) (Fig. 3E). There is no interaction (F_(4,56)_ = 1.25, p = 0.301). We could not detect changes between EphB2^lacZ/lacZ^ and the littermate wildtype mice in the number of immature neurons stained for DCX (EphB2^lacZ/lacZ^ (n = 9) and wildtype mice (n = 7), p = 0.681)) (Fig. 3F) or mature neurons stained for NeuN (EphB2^lacZ/lacZ^ (n = 9) and wildtype mice (n = 7), p = 0.791)) (Fig. 3G). Thus, activation of EphB2 forward signaling increases the number of immature and mature cells, as described above, but impairing EphB2 forward signaling does not affect the number of these cells. This leads to the conclusion that EphB2 activation stimulates NSCs to produce new neurons and that EphB2 impairments can be compensated. In addition, EphB2 dysfunction that causes long-term memory impairment is not mediated by defects in neurogenesis.

## Discussion

In this study, we show that activation of EphB2 forward signaling in neural stem cells in the BLA leads to the formation of more immature and mature neurons in the BLA. Impairing EphB2 forward signaling does not affect the number of immature and mature neurons in the BLA. Activation of EphB2 forward signaling in the BLA that leads to an increase in the formation of mature neurons enhances auditory, but not contextual, long-term fear memory. The results show that it is possible to facilitate the formation of new neurons in the adult brain by activating EphB2 forward signaling and that such an increase in neuronal content in the BLA can enhance the future formation of BLA-dependent memories. In addition, we can conclude from these experiments that the effect of optoEphB2 is on the association between the CS and US and not on the shock or its association with the context because the activation of optoEphB2 does not affect contextual fear conditioning.

The activation of EphB2 forward signaling is sufficient to increase the level of mature neurons in BLA neurons and enhance fear memory formation. The enhancement of fear memory formation suggests that the new neurons integrate appropriately into the amygdala circuitry that mediates the CS and US association in fear conditioning. Moreover, it exhibits a possible principle that increasing the number of mature neurons in a brain area can facilitate its performance. Indeed, it has been shown that newly generated hippocampal dentate granule neurons, for example, are recruited by afferent activity and display increased excitability, enhanced activity-dependent plasticity, and a high rate of synaptogenesis, and once they become mature show all the hallmarks of neurons generated during development [26]. In addition, newly generated neurons in the adult hippocampus are increased by training that leads to the formation of a hippocampal-dependent memory and potentially participate in it [27]. Further studies will be needed to understand how these newly formed neurons in the BLA integrate into the functional circuits.

Most of the observations of neurogenesis were in the subventricular zone where NSCs give rise to cells that migrate to the olfactory bulb before they differentiate into different types of neurons and in the subgranular zone of the hippocampal dentate gyrus (DG), where NSCs produce glutamatergic neurons that integrate into the granule cell layer [2]. However, neurogenesis was also been detected in the amygdala [28]. For example, it was shown that neurogenic precursor cells are present in the mouse adult BLA, and can generate functional interneurons [7], in rat amygdala [6], in the amygdala of adult primates [5], and postnatal neurogenesis in the human amygdala was also detected [8]. Our study shows that further EphB2 activation-increased neurogenesis in the BLA facilitates memory formation.

Interestingly, although activation of EphB2 in NSCs in the BLA facilitated the formation of immature and mature neurons in the BLA we found that abolishing EphB2 forward signaling has no effect on immature and mature neurons in the BLA. However, in the hippocampal subgranular zone (SGZ) reduced DCX-positive late-stage progenitors are observed in the EphB2^−/−^ and EphB2^lacZ/lacZ^ mice [11]. It is therefore possible that the amygdala and hippocampus have different molecular mechanisms that govern neurogenesis. This observation also suggests that there are other compensatory molecules whose activity can still lead to normal neurogenesis in the BLA. One possibility is that reverse signaling, that is intact in the EphB2^lacZ/lacZ^ mice, is effective in maintaining the basal level of neurogenesis in the amygdala but not in the hippocampus. It is possible that EphB2 reverse signaling from cells that are in contact with the NSCs in the amygdala is needed for such maintenance. Another possibility is that the basolateral amygdala contains other proteins that affect neurogenesis. For example, sonic hedgehog is found in the basolateral amygdala and is involved in neurogenesis and fear memory [4, 29]. We suggest that a reduction in signaling from EphB2 does not go below the signal threshold needed for neurogenesis. On the other hand, increasing the signaling by optoEphB2 leads to an increase in the signaling pathways that facilitate neurogenesis.

The fact that EphB2^lacZ/lacZ^ mice are impaired in fear memory but have no effect on the number of mature neurons does not contradict the findings in the optoEphB2 experiment for several reasons: (1) Other signaling molecules, than these localized at the NSCs and affected by optoEphB2, may be affected by EphB2 dysfunction in mature neurons in the EphB2^lacZ/lacZ^ and are also needed for fear memory formation (such as glutamate receptors in the synapse). These molecules can be the major effectors of memory impairments in the EphB2^lacZ/lacZ^. (2) Another difference that should be considered between the optoEphB2 and EphB2^lacZ/lacZ^ experiments is that the former affects EphB2 signaling in adults only whereas the latter affects the brain also during development and may set differences in cellular and molecular compositions.

There is some reduction in the level of general freezing in mice in the optoEphB2 experiment compared to the EphB2^lacZ/lacZ^ experiment in both experimental and control groups. This can be attributed to the fact that the optoEphB2 animals underwent surgical procedures which can reduce freezing in general or to the fact that we used different lines of animals. However, since the experimental and control groups in Figs. 2 and 3 went through the exact procedures the groups within the experiments can be compared with each other.

EphB2 can enhance fear memory formation if it is activated in progenitor (current study) or mature neurons [14] however the mechanisms are probably different. In mature neurons, the activation of EphB2 is associated with changes in synaptic efficacy especially an increase in glutamate transmission and morphogenesis of dendritic spines^e.g.^[30, 31]. These synapses are not found in progenitor cells and immature neurons where EphB2 exerts its effects in our current experiment. Thus, EphB2 probably activates different signaling pathways. It is possible therefore that the same molecule, e.g. EphB2, can have a different effect on cells as shown, for example, during brain development, and in different cell types.

It has been shown that learning, an enriched environment, and physical activity stimulate changes in the composition of other cell types in the amygdala such as alterations in the number of microglia [4, 32, 33]. It would be of interest to examine whether such changes in cell composition is mediated by alterations in EphB2 activities.

Impaired neurogenesis is involved in mental disorders [1]. For example, reduced and impaired neurogenesis is associated with depression, anxiety [34], schizophrenia [35, 36], and with Fragile X syndrome [37]. Reduced neurogenesis may also be associated with neurodegenerative diseases such as Parkinson’s disease, Huntington’s disease, and Alzheimer’s disease [38]. It would be therefore very useful to treat these patients with treatments that lead to an increase in neurogenesis. The current study shows that EphB2 forward signaling can lead to an increase in neurogenesis and can serve as a potential treatment for these mental and neurodegenerative disorders.

## Data Availability

The datasets generated and analyzed during the current study are available from the corresponding author on reasonable request.
